# Prevalence of *Salmonella* spp. in Packed and Unpacked Red Meat and Chicken in South of Tehran

**DOI:** 10.5812/jjm.9254

**Published:** 2014-04-01

**Authors:** Mohammad Mehdi Soltan Dallal, Mohammad kazem Sharifi Yazdi, Nima Mirzaei, Enayat Kalantar

**Affiliations:** 1 Food Microbiology Research Center, Tehran University of Medical Sciences, Tehran, IR Iran; 2 Division of Microbiology, Department of Pathobiology, School of Public Health, Tehran University of Medical Sciences, Tehran, IR Iran; 3 Zoonosis Research Centre, Tehran University of Medical Sciences, Tehran, IR Iran; 4 Department of Medical Laboratory Sciences, School of Paramedicine, Tehran University of Medical Sciences, Tehran, IR Iran; 5 Department of Microbiology, Science and Research Branch, Islamic Azad University, Tehran, IR Iran; 6 Department of Microbiology, Faculty of Medicine, Alborz University of Medical Sciences, Karaj, IR Iran

**Keywords:** *SopD Protein, Salmonella*, Meat Products, Chicken, Food Quality

## Abstract

**Background:**

Despite of the advances in infectious diseases prevention and food technology, food-borne diseases are considered major problems in developed and developing countries. Meat plays a key role in transferring zoonotic diseases to human.

**Objectives:**

This study was conducted in south of Tehran, Iran, to investigate the prevalence rate of *Salmonella* spp. in packed and unpacked red meat and chicken.

**Materials and Methods:**

A total of 379 packed and unpacked samples including 189 red meat and 190 chicken samples were collected randomly. From each sample, 25 g was separated and treated with 225 mL of buffered peptone water, homogenized and incubated at 37°C for 24 hours. Samples were enriched using Rappaport-Vassiliadis broth and then streaked onto Hektoen enteric agar.

**Results:**

Totally, 86 out of 190 chicken and 38 out of 189 red meat samples were contaminated with *Salmonella* spp. The most isolated serotypes were *Salmonella thompson* (67.7%), *S. heaardt* (6.5%), *S. enteritidis* (4.8%), and *S. veyle* (4%), respectively. In general, the rate of chicken contamination was higher than meat, as 43.3% of packed and 46% of unpacked chicken samples were contaminated.

**Conclusions:**

These results confirmed the pervious findings, stating that proper packaging of meat products can effectively decreases the rate of microbial contaminations.

## 1. Background

Foodborne diseases are one of the serious problems in developed and developing countries. Every year, more than 100 million people are afflicted by foodborne and waterborne diseases in the world, especially people with immune system deficiency and malnutrition (1, 2). Different species of *Salmonella*, *Listeria* and *Yersinia* are related to the diseases, which can be transmitted through consuming contaminated fish, dairy products, vegetables and meat (3, 4).

Epidemiological studies have shown that foods of animal origin are among the most important sources of foodborne diseases (5, 6). Food products are usually contaminated with pathogens during the production, processing, distributing and retailing in the market (7). Different studies showed that the frequencies of beef contaminations with human pathogens such as *Salmonella* were not the same (8-10).

Salmonellosis is the major cause of foodborne infections and the second-most common foodborne illness after *Campylobacter* infection (11). *Salmonella* infection in human is often resulted from ingestion of contaminated foods such as beef, pork, egg, milk, seafood, and fresh products (11-15). Pathogens can survive in the food products, especially in meat, until distributed in the markets (16, 17). One of the best methods to prevent food products contamination is packaging. In the industrialized countries, food products are mostly distributed in the form of packages. In Iran, some food products such as chicken and beef are traditionally distributed unpackaged. Meat products are one of the most consumed foods; hence, they have a key role in transmission of *Salmonella* to humans. There are annually thousands of reported cases of foodborne diseases related to *Salmonella* in Iran. Many of *Salmonella* specie isolated from meat products are multidrug resistant strains, which can cause serious problems (18, 19).

## 2. Objectives

The aim of this study was to compare the contamination rate of *Salmonella* in packaged and unpackaged chicken and beef products as well as the effect of packaging on contamination prevention.

## 3. Materials and Methods

### 3.1. Sample Collection

A total of 379 samples, including 189 beef and 190 chicken samples were collected from different stores in Tehran. All samples were kept at 4°C before and during transferring to the laboratory.

### 3.2. Isolation, Identification and Serotyping of *Salmonella*

Identification of *Salmonella* was performed according to ISO-6575 (20). From each sample, 25 g was placed in a sterile stomacher bag and 225 mL buffered peptone water, pH 7.0 (Merck, Germany) was added to it. Samples were then homogenized using a stomacher for at least 2 minutes, followed by incubation at 37°C for 24 hours. Afterwards, 0.1 mL of pre-enriched broth was transferred into 10 mL of Rappaport-Vassiliadis medium (Oxoid, UK) and incubated at 42°C for 24 hours.

The enriched samples were then plated onto Hektoen enteric agar (Sigma, USA) and incubated at 37°C for 24 hours. Colonies on Hektoen enteric agar were then identified using biochemical tests such as oxidase reaction, acid production from manitol, ONPG test, H_2_S and indole production, and urease and lysine decarboxylase activity. For final identification and verification, isolated strains were cultured on differential media such as Simmon’s citrate agar, urea broth, lysine iron agar, methyl red and sulfide indole motility (SIM), and then incubated at 37°C for 24 hours.

Serotyping was performed according to Kaufmann-White scheme using O antisera (Difco, USA) (21) and flagellar antigens were detected by a technique of utilizing microtitre plates (22). Putative *Salmonella* isolates were transferred to Razi Vaccine and Serum Research Institute for serotyping.

### 3.3. Statistical Analysis

Statistical analysis was performed using SPSS software version 19. The P values < 0.05 were considered statistically significant.

## 4. Results

Out of 379 samples, 134 (32.7%) were contaminated (Table 1) and 13 serotypes were identified (Table 2). The most isolated serotypes were *S. thompson* (67.7%), *S. haardt* (6.5%) and *S. veyle* (4.8%), respectively (Table 2). Of *S. thompson* serotype, 65 were isolated from beef and 19 from chicken. Rates of isolated *Salmonella* spp. from unpackaged and packaged beef and chicken samples were 22.3%, 16.4%, 46%, 43.3%, respectively (Table 3). Isolation rate of *Salmonella* spp. from unpackaged chicken was 46%, which was significantly higher than unpackaged beef with the rate of 22.3%. The statistical analysis difference rate among different sources of chicken and beef *Salmonella* spp. isolates was P < 0.05. Serotypes were more variable in unpackaged samples (Table 4). The contamination rates of samples were not related to the collection region.

The most isolated serotype from the packaged beef and chicken was *S. thomson* with the rate of 4.8% and 28.6%, respectively. *S. enteritidis* (16.7%), *S. haardt* (12.5%) and *S. virginia* (33.3%) were detected in the packaged chicken samples (Table 5) and *S. anatum* and *S. meleagridis* (100%) were detected only in the packaged samples, *S. paratyphi* C and *S. kentucky* (100%) were detected only in the unpackaged samples (Table 4). 

The most isolated serotypes in the unpackaged beef and chicken samples were *S. thampson*, *S. paratyphi*, *S. haard*, *S. enteritidis*, and *S. virigina* was detected in the unpackaged chicken samples. *S. voyle* (60%), *Salmonella *group II (100%) and *S. kentucky* (100%) were isolated from the unpackaged beef samples. *Salmonella *group F, *S. meleagrides* were not isolated from the unpackaged samples (Table 5). 

**Table 1.** Prevalence of *Salmonella* spp. in Beef and Chicken Samples ^a^

Type of Sample	Positives	Negative	Total
**Chicken**	86 (45)	104 (55)	190 (100)
**Beef**	38 (20.2)	151 (79.8)	189 (100)
**Total**	124 (32.7)	255 (67.3)	379 (100)

^a^ Data are presented as No. (%).

**Table 2.** Distribution of *Salmonella* Serotypes in Beef and Chicken Samples ^a^

Serotype	Type of Samples		
	Beef	Chicken	Total
***S. thompson***	19 (50)	65 (75.6)	84 (67.7)
***S. paratyphi C***	1 (2.6)	2 (2.3)	3 (2.4)
***S. anatum***	1 (2.6)	0 (0)	1 (0.8)
***S. veyle***	5 (13.2)	0 (0)	5 (4)
***S. enteritidis***	1 (2.6)	5 (5.8)	6 (4.8)
***S. haardt***	2 (5.3)	6 (7)	8 (6.5)
***S. virginia***	0 (0)	3 (3.5)	3 (2.4)
*** Salmonella *group II**	1 (2.6)	0 (0)	1 (0.8)
***S. meleagridis***	2 (5.3)	0 (0)	2 (1.6)
***S. typhimurium***	2 (5.3)	1 (1.2)	3 (2.4)
***S. untypable***	2 (5.3)	4 (4.7)	6 (4.8)
***S. kentucky***	1 (2.6)	0 (0)	1 (0.8)
***Salmonella *group F**	1 (2.6)	0 (0)	1 (0.8)
**Total**	38 (100)	86 (100)	124 (100)

^a^ Data are presented as No. (%).

**Table 3.** Distribution of *Salmonella* Serotypes in the Packaged and Unpackaged Chicken and Beef Samples ^a^,^b^

Type of Sample	Positives	Negative	Total
**Packaged beef**	11 (16.4)	56 (83.6)	67 (100)
**Unpackaged beef**	27 (22.3)	93 (77.7)	122 (100)
**Packaged chicken**	29 (43.3)	38 (56.7)	67 (100)
**Unpackaged chicken**	57 (46)	66 (45)	123 (100)
**Total**	124 (32.7)	223 (58.8)	379 (100)

^a^ Data are presented as No. (%).

^b^ x2 = 6.91, d. f. = 3, P = 0.75.

**Table 4.** Distribution of *Salmonella* Serotypes in the Packaged and Unpackaged Samples ^a^

Serotype	Type of Sample		
	Packaged	unpackaged	Total
***S. thompson***	28 (70)	56 (66.7)	84 (67.7)
***S. paratyphi C***	0 (0)	3 (3.6)	3 (2.4)
***S. anatum***	1 (2.6)	0 (0)	1 (0.8)
***S. veyle***	2 (5)	3 (3.6)	5 (4)
***S. enteritidis***	1 (2.6)	5 (5.8)	6 (4.8)
***S. haardt***	1 (2.6)	7 (8.3)	8 (6.5)
***S. virginia***	1 (2.6)	2 (2.4)	3 (2.4)
***Salmonella *group II**	0 (0)	1 (1.2)	1 (0.8)
***S. meleagridis***	2 (5)	0 (0)	2 (1.6)
***S. typhimurium***	0 (0)	3 (3.6)	3 (2.4)
***S. untypable***	3 (7.5)	3 (3.6)	6 (4.8)
***S. kentucky***	0 (0)	1 (1.2)	1 (0.8)
***Salmonella *group F**	1 (2.6)	0 (0)	1 (0.8)
**Total**	40 (100)	84 (100)	124 (100)

^a^ Data are presented as No. (%).

**Table 5.** Distribution of *Salmonella* Strains in the Packaged and Unpackaged Beef and Chicken Samples ^a^

Serotype	Chicken		Beef	
	Packaged	Unpackaged	Packaged	Unpackaged
***S. thompson*** ** (n = 84)**	24 (28.6)	41 (48.8)	4 (4.8)	15 (17.9)
***S. paratyphi C*** ** (n = 3)**	0 (0)	2 (66.7)	0 (0)	1 (33.3)
***S. anatum*** ** (n = 1)**	0 (0)	0 (0)	0 (0)	1 (100)
***S. veyle*** ** (n = 5)**	0 (0)	0 (0)	2 (40)	3 (60)
***S. enteritidis*** ** (n = 6)**	1 (16.7)	4 (66.7)	0 (0)	1 (16.7)
***S. haardt*** ** (n = 8)**	1(12.5)	5 (62.5)	0 (0)	2 (25)
***S. virginia*** ** (n = 3)**	1 (33.3)	2 (66.7)	0 (0)	0 (0)
***Salmonella *group II** ** (n = 1)**	0 (0)	0 (0)	0 (0)	1 (100)
***S. meleagridis*** ** (n = 2)**	0 (0)	0 (0)	2 (100)	0 (0)
***S. typhimurium*** ** (n = 3)**	0 (0)	1 (33.3)	0 (0)	2 (66.7)
***S. untypable*** ** (n = 6)**	2 (33.3)	2 (33.3)	1 (16.7)	1 (16.7)
***S. kentucky*** ** (n = 1)**	0 (0)	0 (0)	0 (0)	1 (100)
***Salmonella *group F** ** (n = 1)**	0 (0)	0 (0)	1 (100)	0 (0)

^a^ Data are presented as No. (%).

## 5. Discussion

Salmonellosis is one of the most important foodborne diseases (23). High prevalence of *Salmonella* species in chicken and beef samples obtained in this study was similar to the previous studies (11, 24-26). *Salmonella* spp. infections are usually caused by handling or consuming contaminated food products (27-30). In this study, the prevalence of *Salmonella* spp. in chicken meat samples was higher than beef samples. After death, the level of pH is reduced in animal tissues, leading to the decrease of bacterial growth in beef samples. A few previous studies have shown that bacteria normally grow more slowly in meat products with low pH levels (31). 

Serotypes such as *S. thampson*, *S. typhimurium*, *S. enteridis*, *S. infantis*, *S. paratyphi* B were isolated from chicken and beef samples in the previous studies (32). The most isolated serotypes in this study were *S. thompson*, *S. paratyphi C*, *S. enteridis*, *S. infantis*, *S. haardt*, and *S. typhimurium*, which was similar to other finding (26). Isolation of invasive serotypes such as *S. typhimurium* indicates the public health significance and may pose health hazards, especially if chicken is consumed undercooked or cross-contamination occurs in kitchen during the meal preparation. The dominated serotype was *S. thampson*, which might be due to cross-contamination during product handling and distribution. Infection with *S. thampson* causes diarrhea, nausea and vomiting in humans as well as several diseases in immune-compromised individuals (33). 

Contamination rates of the unpackaged samples with different serotypes of *Salmonella* spp. were higher compared with the packaged ones in both chicken and beef samples. Moreover, *Salmonella* serotypes were detected significantly higher in the unpackaged chicken compared with unpackaged beef; this might be due to specific physiological features of the beef tissue. We detected *Salmonella* serotypes more frequently during June, July, September and August, the hottest months of the year in Tehran. Previous studies also showed higher levels of *Salmonella* serotypes detection during summer (34).
